# The Neuraminidase Stalk Deletion Serves as Major Virulence Determinant of H5N1 Highly Pathogenic Avian Influenza Viruses in Chicken

**DOI:** 10.1038/srep13493

**Published:** 2015-08-26

**Authors:** Olga Stech, Jutta Veits, El-Sayed M. Abdelwhab, Ute Wessels, Thomas C. Mettenleiter, Jürgen Stech

**Affiliations:** 1 Institute of Molecular Virology and Cell Biology, Friedrich-Loeffler-Institut, Federal Research Institute for Animal Health, Südufer 10, 17493 Greifswald-Insel Riems, Germany

## Abstract

Highly pathogenic avian influenza viruses (HPAIV) cause devastating losses in gallinaceous poultry world-wide and raised concerns of a novel pandemic. HPAIV develop from low-pathogenic precursors by acquisition of a polybasic HA cleavage site (HACS), the prime virulence determinant. Beside that HACS, other adaptive changes accumulate in those precursors prior to transformation into an HPAIV. Here, we aimed to unravel such virulence determinants in addition to the HA gene. Stepwise reduction of HPAIV genes revealed that the HPAIV HA and NA form a minimum set of virulence determinants, sufficient for a lethal phenotype in chicken. Abolishing the NA stalk deletion considerably reduced lethality and prevented transmission. Conversely, the analogous stalk deletion reconstructed in the NA of an LPAIV reassortant carrying only the HPAIV HA resulted in 100% lethality both after primary and contact infection. Remarkably, the unmodified LPAIV NA with its long stalk, when exclusively introduced into the H5N1 HPAIV, still enabled high virulence and efficient transmission. Therefore, irrespective of an NA stalk deletion, minor virulence determinants in addition to the essential polybasic HACS contribute to high virulence, whereas the NA stalk deletion alone may serve as major virulence determinant.

Highly pathogenic avian influenza viruses (HPAIV) cause devastating losses in poultry world-wide and raise concerns about a novel pandemic due to repeated zoonotic transmissions to humans^1^. These strains develop from low-pathogenic precursors specifying the hemagglutinin (HA) serotypes H5 or H7. HPAIV carry a polybasic HA cleavage site (HACS)^2^,^3^,^4^,^5^ whereas the HA of all low-pathogenic avian viruses (LPAIV) and human influenza viruses carry a mono- or dibasic motif^6^,^7^,^8^,^9^,^10^. In HPAIV, the polybasic HACS is the prime virulence determinant essential for high pathogenicity in chicken; its conversion to a monobasic motif renders the virus low-pathogenic^9^,^11^. Conversely, introduction of a polybasic HACS into LPAIV of several HA serotypes does not necessarily result in high virulence^12^,^13^,^14^,^15^. Interestingly, four natural H5 strains with polybasic HACS but low pathogenicity in chickens were found^16^. Furthermore, LPAIV single-gene reassortants carrying an HPAIV HA display variable levels of virulence^12^,^13^,^17^. The reciprocal gene constellation, i.e. an LPAIV HA with engineered polybasic HACS plus the other seven gene segments from an HPAIV caused highly pathogenic phenotypes even in case of a nonH5/H7 HA^12^,^13^,^17^. Moreover, specific gene constellations involving PB2, PB1, and NP may enable high virulence^18^. Taken together, those findings revealed that, besides the essential polybasic HACS, additional virulence determinants reside in HA and the other seven genes. Such additional adaptive changes might accumulate in the low-pathogenic precursors during their circulation in gallinaceous poultry prior to an HPAIV outbreak^2^,^3^,^4^,^5^. In this study, we aim to elucidate those additional virulence determinants of all nonHA genes.

## Results

### Selection reveals that HPAIV PB2, HA, NP, NA, and M confer high virulence

To reveal HPAIV gene constellations sufficient for high virulence, we performed selection experiments with the genes of HPAIV A/Swan/Germany/R65/2006 (H5N1) (R65) versus those of LPAIV A/Teal/Germany/Wv632/2005 (H5N1) (TG05). To this end, we co-transfected plasmids encoding the R65 and TG05 genes, used the resulting supernatants for infection of chickens, took oral swabs and screened them for reassortant genotypes. Since we already could demonstrate that the introduction of the R65 HA into the genetic background of TG05 (reassortant TG05-HA_R65_) leads to 100% morbidity and 30% lethality in chicken^12^, we omitted the TG05 HA but retained the R65 HA.

**Figure 1:**
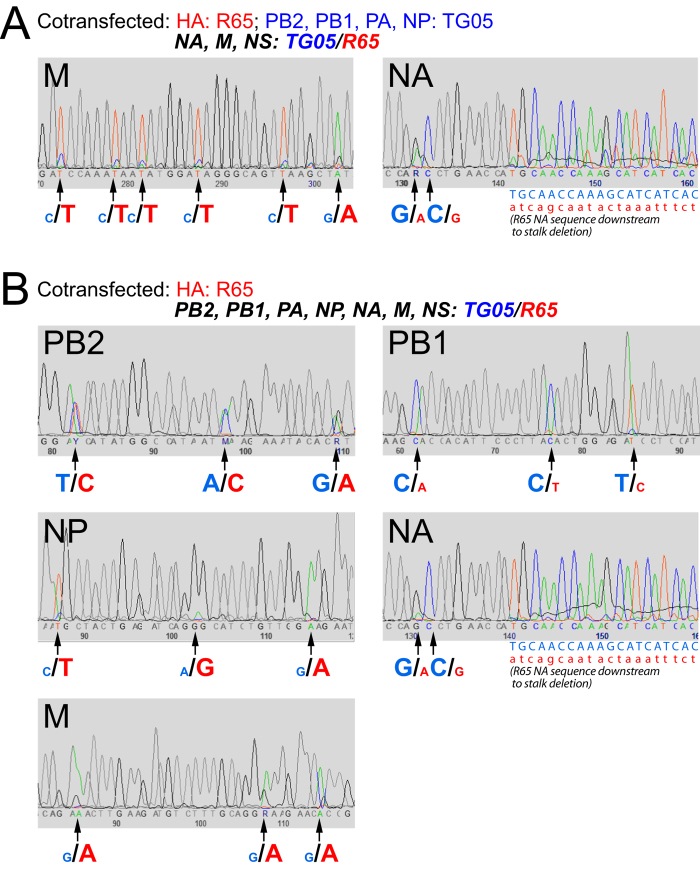
Simultaneous presence of TG05 and R65 genes determined by Sanger sequencing of supernatants following plasmid co-transfection to obtain mixtures of random reassortants. (**A**) The TG05 polymerase gene plasmids (PB2, PB1, PA, NP) and the R65 HA plasmid were co-transfected together with mixtures of the NA, M, and NS plasmids originating both from TG05 and R65. (**B**) The TG05 plasmids except the HA plasmid were co-transfected together with all eight R65 plasmids.

**Figure 2:**
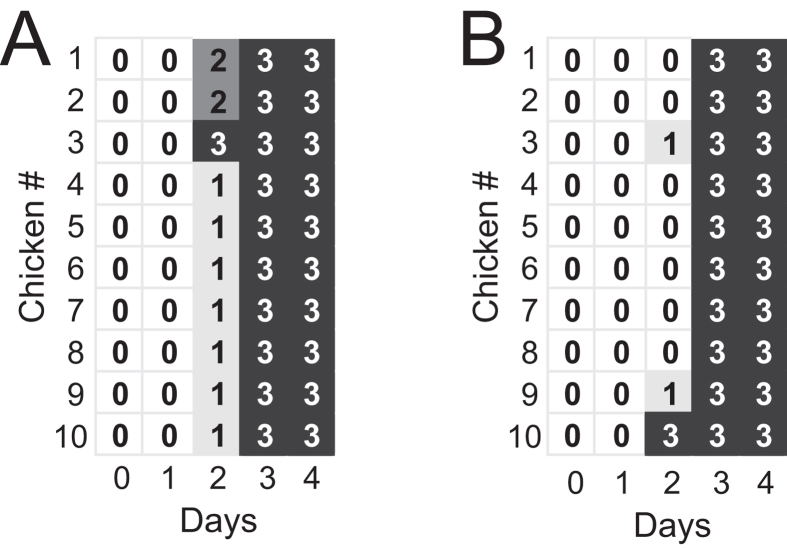
High lethality following oculonasal infection of chickens with 600 μl co-transfection supernatants. Daily clinical score: 0: healthy, 1: ill, 2: severely ill or 3: dead. (**A**) Birds infected with supernatant from co-transfection of the TG05 polymerase gene plasmids (PB2, PB1, PA, NP), the R65 HA plasmid and mixtures of the NA, M, and NS plasmids originating both from TG05 and R65. (**B**) Birds infected with supernatant from co-transfection of the TG05 plasmids except the HA plasmid and all eight R65 plasmids.

First, to narrow selection to the small R65 genes (NA, M, and NS), we co-transfected the TG05 polymerase gene plasmids (PB2, PB1, PA, NP) and the R65 HA plasmid together with mixtures of the NA, M, and NS plasmids originating both from TG05 and R65. Sanger sequencing of the post-transfection supernatant indicated the simultaneous presence of the NA and M genes from both TG05 and R65 plus the presence of the TG05 NS gene (Fig. 1a). Oculonasal infection of ten chickens with this post-transfection mixture resulted in death of all animals by day 3 (Fig. 2a). Genotyping of 30 isolated plaques from oral swabs taken on days 2 and 3 from three animals (#1, #3 (which died and therefore was swabbed on day 2 only), and #4; ten plaques each) revealed a preference for the R65 NA and M genes (except one plaque from animal #4 containing the R65 NA but the TG05 M) or, in another animal (#2 from which only one plaque could be isolated), the presence of the R65 NA gene only.

**Figure 3:**
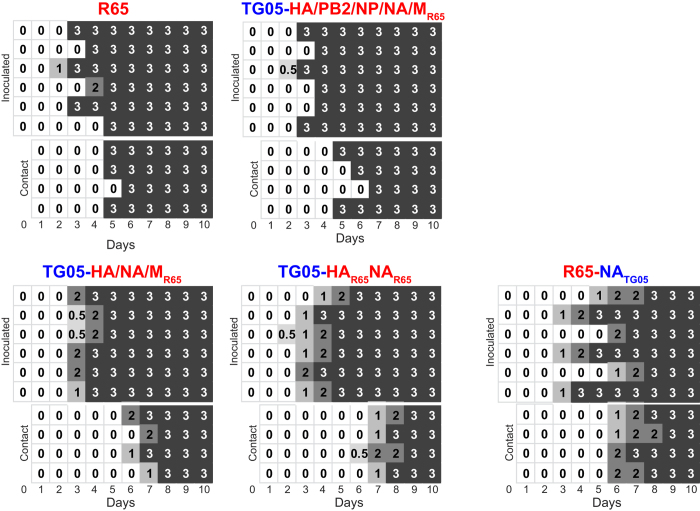
Minimal R65 gene constellations sufficient for a lethal phenotype and transmission in chicken. We infected the birds oculonasally with 10^5^ pfu virus and added bystander animals on day 1 p. i. Daily clinical score: 0: healthy, 1: ill, 2: severely ill or 3: dead.

To allow for selection of *all* R65 genes, we then co-transfected the TG05 plasmids except the HA plasmid together with all eight R65 plasmids. Sanger sequencing of the post-transfection supernatant revealed mixtures of the PB2, PB1, NP, NA, and M genes, whereas PA and NS appeared to originate from TG05 predominantly (Fig. 1b). Oculonasal infection of chickens with the post-transfection supernatant resulted in death of all ten animals on day 3 post infection except animal #10 which already succumbed to death on day 2 (Fig. 2b). We then isolated plaques from oral swabs taken on day 2 from animals #2 and #10. The genotyping of five plaques from animal #2 and one from animal #10 indicated the presence of the TG05 PB1, PA, and NS1 genes and (in addition to the HA) of the PB2, NP, NA, M genes from R65. To investigate whether this gene composition confers high virulence, we reconstituted the reassortant TG05-HA/PB2/NP/NA/M_R65_ by reverse genetics to infect chickens. All directly infected animals died on days 3–4 and the contact animals succumbed to death on days 5–7, indicating a virulence equivalent to that of R65 (Fig. 3). Taken together, the screening for random reassortant genotypes shed by chickens infected with supernatants after co-transfection with TG05/R65 plasmid mixtures indicated that, in addition to the R65 HA, the PB2, NP, NA, and M form an optimal gene combination enabling high virulence like the parent virus R65.

### Stepwise reduction of HPAIV genes reveals that R65 HA plus NA are sufficient for high virulence and transmission

To identify the specific HPAIV genes conferring high virulence, we generated tailored reassortants from HPAIV R65 and LPAIV TG05 by reverse genetics^19^. Since introduction of the R65 HA into the genetic background of TG05 resulted in 100% morbidity and 30% lethality in chicken (reassortant TG05-HA_R65_ in^12^), we stepwise replaced several TG05 genes in TG05-HA_R65_ by their R65 counterparts yielding these reassortants: TG05-HA/PB2/PB1/PA/NP_R65_ contains the TG05 NA, M, and NS genes and the R65 HA plus polymerase complex genes (PB2, PB1, PA, and NP), whereas reassortant TG05-HA/NA/M/NS_R65_ carries the HA plus the NA, M, and NS genes from R65 but the polymerase complex genes of TG05. Oculonasal inoculation of ten chickens with 10^5^ pfu of TG05-HA/PB2/PB1/PA/NP_R65_ resulted in a lethality of 30% like TG05-HA_R65_^12^ indicating that the R65 polymerase complex alone does not confer high virulence. In contrast, the reciprocal “small” gene reassortant TG05-HA/NA/M/NS_R65_ exhibited 100% lethality resembling R65. Furthermore, the 2.9 days (d) mean death time (MDT) of TG05-HA/NA/M/NS_R65_ is close to that of R65 with 3.8 d (range 3–5 d, six animals) (Table 1, Fig. 3), indicating that the R65 HA, NA, M or NS genes are sufficient for a highly pathogenic phenotype.

To identify which of the R65 NA, M or NS gene(s) is responsible for high virulence, we infected chickens with the three triple reassortants TG05-HA/NA/M_R65_, TG05-HA/NA/NS_R65_, TG05-HA/M/NS_R65_, or the double reassortant TG05-HA_R65_NA_R65_. TG05-HA/NA/M_R65_ and TG05-HA/NA/NS_R65_ displayed 100% lethality with increased MDT of 3.6 d and 5.2 d, respectively, suggesting a decrease in virulence compared to the quadruple reassortant TG05-HA/NA/M/NS_R65_ (MDT 2.9 d) (Table 1). In contrast, replacement of the R65 NA gene by that of TG05 as in TG05-HA/M/NS_R65_ resulted in death of only three of eight animals similar to TG05-HA_R65_^12^, indicating a crucial role of the NA for high virulence in R65 (Table 1). Correspondingly, the HA/NA reassortant TG05-HA_R65_NA_R65_ (which differs from TG05-HA_R65_ only by the R65 NA) displays 100% lethality, although at a prolonged MDT of 5.9 d (range 5–7 d), proving that introduction of the R65 NA gene into TG05-HA_R65_ resulted in a highly pathogenic phenotype (Table 1). Since M, NS or the polymerase complex contribute to the high virulence of R65 to a very limited extent only in the presence of the R65 NA, the NA represents a major virulence determinant.

To investigate the impact of the R65 NA and M on virus transmission, we infected six chickens with the reassortants TG05-HA/NA/M_R65_ or TG05-HA_R65_NA_R65_ and added four contact animals after one day. In both groups, all primarily infected animals died on days 4 to 6. All contact animals succumbed to death on days 7 to 8 with TG05-HA/NA/M_R65_ and days 8 to 9 with TG05-HA_R65_NA_R65_ (Fig. 3). To investigate whether all other R65 genes combined but except the NA can confer high virulence, we replaced the NA in R65 by that of TG05 resulting in the reassortant R65-NA_TG05_. Oculonasal infection of chickens resulted in 100% mortality both in the primarily infected and the contact animals (Fig. 3), in contrast to TG05-HA/M/NS_R65_ displaying a mortality of 37.5% (Table 1). Thus, in the absence of the R65 NA, the HA, M, and NS can only confer a highly pathogenic phenotype in conjunction with the polymerase genes of R65. On the other hand, R65 HA and NA alone are sufficient for high virulence and transmission.

### NA stalk deletion plus polybasic HACS form a minimal set of virulence determinants

**Figure 4:**
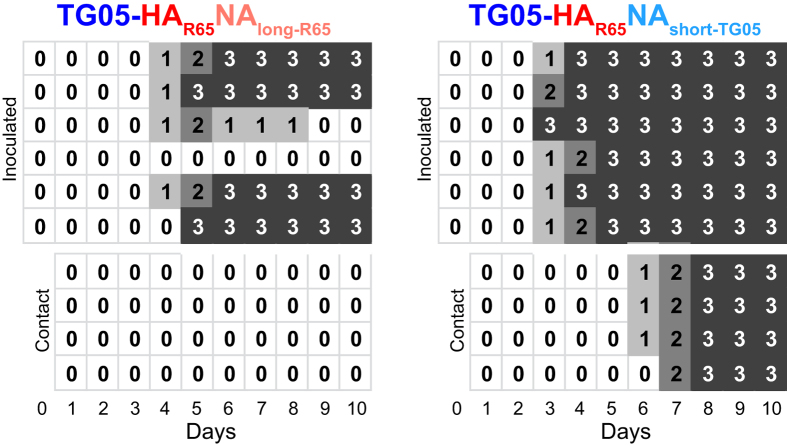
NA stalk deletion is crucial for transmission among chickens. We infected chickens oculonasally with 10^5^ pfu virus and added bystander animals on day 1 p. i. Daily clinical score: 0: healthy, 1: ill, 2: severely ill or 3: dead.

After having shown that the R65 HA and NA alone are sufficient for a highly pathogenic phenotype, we addressed the question whether the stalk deletion of the R65 NA, increasingly found in contemporary H5N1 HPAIV^20^, is crucial for high virulence and virus transmission. To this end, we generated two different NA mutant viruses. Removal of amino acids (aa) 49 to 68 in the TG05 NA stalk region resulted in the same stalk deletion as in the NA of R65; this NA mutant plasmid was used to rescue TG05-HA_R65_NA_short-TG05_. For a mirror-imaged virus mutant, we inserted amino acids 49–68 from the TG05 NA into the stalk region of the R65 NA to rescue TG05-HA_R65_NA_long-R65_. After infection of six chickens with 10^5^ pfu and addition of 4 contact animals one day later, TG05-HA_R65_NA_long-R65_ caused death in four of six animals and no symptoms in the contact animals (Fig. 4). However, TG05-HA_R65_NA_short-TG05_ in striking contrast to TG05-HA_R65_ (30% lethality) (Table 1), displayed 100% lethality both in the primarily infected and the contact animals demonstrating high virulence and efficient transmission (Fig. 4). Therefore, in R65 the NA stalk deletion is a major but non-essential virulence determinant which, together with R65 HA carrying a polybasic cleavage site, is sufficient to confer high virulence on TG05.

## Discussion

HPAIV evolve from low-pathogenic precursors by acquisition of a polybasic HACS^4^ which is not per se sufficient for high virulence^12^,^13^,^14^,^15^,^16^,^17^. Prior to the emergence of HPAIV, those precursor strains often circulate in gallinaceous poultry thereby accumulating mutational changes^2^,^3^,^4^,^5^. In this study, we aimed to elucidate the genetic determinants which in addition to those of the HA gene facilitate the transformation of LPAIV into HPAIV by two approaches.

First, to reveal minimal gene constellations conferring high virulence, we selected random reassortants by co-transfection of plasmids from both the HPAIV R65 (H5N1) and LPAIV TG05 (H5N1) followed by infection of chickens. In contrast to the classical technique to obtain reassortants by double-infection^21^,^22^, this approach allowed us to exclude specific viral genes by omitting the respective plasmids. Infection of chickens with the supernatant after co-transfection of plasmids coding for all eight R65 but seven TG05 genes without HA resulted in 100% lethality. Orally shed reassortants carried the R65 PB2, NP, HA, NA, and M genes, whereas the PB1, PA and NS genes originated from TG05. Reconstitution of this genotype by reverse genetics led to an HPAIV undistinguishable from R65. This finding suggests that the TG05 PB1, PA, and NS genes do not require further adaptation, whereas a highly pathogenic phenotype is conferred by the R65 HA plus the PB2, NP, NA, and M genes.

Secondly, we investigated specific reassortants from R65 and TG05 in chicken. Stepwise reduction of the R65 genes beginning with reassortant TG05-HA/NA/M/NS_R65_ resulted in prolonged mean death times indicating limited contribution of the R65 polymerase, M, and NS genes to virulence (Table 1). Remarkably, the R65 polymerase and NP, or the M and NS genes on their own were not sufficient for high virulence. Such subtle changes in virulence are likely not reflected by the intravenous pathogenicity index test^23^ since the intravenous inoculation ensures standardization but bypasses infection of the natural target tissues. In contrast, high lethality and efficient transmission of the single-gene reassortant R65-NA_TG05_ demonstrates that beside HA, the R65 polymerase including NP *plus* the M and NS genes yield an HPAIV. On the other hand, high virulence was conferred by the R65 HA and NA genes alone (reassortant TG05-HA_R65_NA_R65_).

We then investigated whether the NA stalk deletion in R65 is crucial for high virulence and transmission to contact animals. Repairing the stalk deletion led to reduced virulence and prevented transmission, whereas introduction of the NA stalk deletion into the TG05 NA as in the mutant TG05-HA_R65_NA_short-TG05_ increased lethality and transmission to 100% compared with its parental virus TG05-HA_R65_ displaying 30% lethality^12^. This finding reveals that in the presence of an HPAIV HA with polybasic HACS, virulence determinants, already present in LPAIV, like the NA stalk deletion^24^,^25^,^26^,^27^ alone may support the transformation to a highly pathogenic phenotype.

Current understanding on functional consequences of the NA stalk deletion is still limited. An elevated V_max_ at a nearly unaffected K_m_ has been demonstrated in case of a small substrate^28^. However, the ability of the virus to elute from agglutinated erythrocytes and to penetrate mucus on cells of mammalian origin is reduced^28^,^29^. Remarkably, the NA stalk deletion is detrimental for virus replication in ducks^30^ but shifts the viral tropism from the intestinal to the respiratory tract in chicken^27^. Correspondingly, the NA stalk deletion is considered an adaptation of low-pathogenic avian strains from waterfowl to domestic poultry, in particular to chicken^27^.

In general, the pairwise comparison of an HPAIV versus its putative low-pathogenic precursor may be limited to only a partial subset of a concealed set of virulence determinants, all together conferring high virulence. Such a complete set may be formed by an NA stalk deletion as a *major* but non-essential plus the polybasic HACS as the prime and *essential* virulence determinant. However, without the NA stalk deletion, a highly pathogenic phenotype is displayed by the reassortant R65-NA_TG05_ which in addition to the R65 HA carries the R65 polymerase and NP genes together with the R65 M and NS genes. Thus, the aa exchanges of those genes can be considered an alternative set of *minor* virulence determinants among which a single exchange contributes to the virulence to only a minute extent. Therefore, HPAIV like R65 may contain at least two different sets of virulence determinants. Taken together, HPAIV require an adapted HA with polybasic HACS as *essential* plus either several adaptive aa changes in polymerase, NP, M, and NA genes as *minor* virulence determinants or an NA stalk deletion as *major* virulence determinant.

## Methods

### Cells and recombinant viruses

Madin-Darby canine kidney (MDCK) cells were cultivated in minimal essential medium containing 10% fetal bovine serum. Plasmids encoding the gene segments of strains A/Swan/Germany/R65/2006 (H5N1) (R65) (Genbank accession numbers DQ464354-DQ464361) and A/Teal/Germany/Wv632/2005 (H5N1) (TG05) (Genbank accession numbers CY061882-9) have been described^12^,^17^. To obtain specific reassortants (1 μg each plasmid) or mixtures of random reassortants (1 μg each TG05 plasmid and 0.1 μg each R65 plasmid^12^) by co-transfection, we rescued recombinant viruses as described and propagated them in 11-day-old embryonated chicken eggs or MDCK cells^9^,^13^. Gene composition of recombinant viruses was verified by Sanger-sequencing of RT-PCR amplicons obtained from viral RNA. Plaque assays were performed on MDCK cells in the presence of 2 μg/ml N-tosyl-L-phenylalanine chloromethyl ketone (TPCK)-treated trypsin (Sigma, Taufkirchen, Germany) or in the absence of any exogenous protease. All viruses were handled under BSL3+ conditions.

### Mutagenesis of NA stalk

To generate a stalk deletion in the TG05 NA (Genbank accession number CY061887) like that in the R65 NA (Genbank accession number DQ464355), we removed nucleotides 165–224 corresponding to aa 49–68. To elongate the stalk region of R65 NA, we inserted nucleotides 165–224 from the TG05 NA gene between nucleotides 164 and 165 of the R65 NA. Those modifications were introduced by site-directed QuikChange mutagenesis (primer sequences available on request).

### Animal experiments

The animal experiments were evaluated by the responsible ethics committee of the State Office for Agriculture, Food Safety and Fishery in Mecklenburg-Western Pomerania (LALFF M-V) and gained governmental approval (registration number LALLF M-V/TSD/7221.3-1.1-018/07). Four- to eight-weeks-old White Leghorn specific-pathogen-free chickens (Lohmann, Cuxhaven, Germany) were infected oculonasally with 10^5^ pfu virus, observed daily for clinical symptoms and classified according to the OIE guidelines^23^ as healthy (0), ill (1) (exhibiting one of the following: respiratory symptoms, depression, diarrhea, cyanosis, edema, or central nervous symptoms), severely ill (2) (severe or more than one of the previously mentioned symptoms), or dead (3). The daily clinical score is the arithmetic mean of individual values. Moribund birds were euthanized according to^23^. The clinical score was calculated by dividing the sum of daily scores (arithmetic mean of the individual scores) by the number of the observation days (10 days). Oral swab samples taken on days 2–3 were subjected to plaque assay on MDCK cells^31^; from picked plaques, viral RNA was isolated and sequences were determined after RT-PCR. For transmission studies, six chickens were infected oculonasally on day 0. The birds were kept in cages of 123 cm length, 65 cm width, and 77 cm height; the bottom was cleaned daily.

## Additional Information

**How to cite this article**: Stech, O. *et al.* The Neuraminidase Stalk Deletion Serves as Major Virulence Determinant of H5N1 Highly Pathogenic Avian Influenza Viruses in Chicken. *Sci. Rep.* **5**, 13493; doi: 10.1038/srep13493 (2015).

## Figures and Tables

**Table 1:** Virulence of parent viruses, TG05/R65 reassortants, and NA stalk variants in chicken.

Virus	Morbidity	Mortality	MDT (d) and Range	Clinical Score
*Parent Viruses*				
TG05^1^	0/10	0/10	n/a	0.00
TG05-HA_R65_^1^	8/10	3/10	n/a	0.90
R65^2^	6/6	6/6	3.8 (3–5)	2.20
*Selected Reassortant*				
TG05-HA/PB2/NP/M/NA_R65_^2^	6/6	6/6	3.5 (3–4)	2.26
*Tailored Reassortants*				
TG05-HA/PB2/PB1/PA/NP_R65_	4/8	3/8	7.3 (6–8)	0.62
TG05/HA/NA/M/NS_R65_	10/10	10/10	2.9 (2–3)	2.54
TG05/HA/NA/M_R65_	10/10	10/10	3.6 (3–4)	2.43
TG05/HA/NA/NS_R65_	10/10	10/10	5.2 (4–6)	1.80
TG05/HA_R65_NA_R65_	8/8	8/8	5.9 (5–7)	1.67
TG05/HA/M/NS_R65_	4/8	3/8	7.0 (6–9)	0.51
R65-NA_TG05_^2^	6/6	6/6	6.2 (4–8)	1.73
*NA Stalk Variants*				
TG05-HA_R65_NA_long-R65_^3^	5/6	4/6	5.5 (5–6)	1.32
TG05-HA_R65_NA_short-TG05_^3^	6/6	6/6	4.2 (3–5)	2.22

Morbidity, mortality and overall clinical score after oculonasal infection with 10^5^ pfu.

^1^Data of this group from previous study^12^.

^2^Identical group as in Fig. 3.

^3^Identical group as in Fig. 4.
